# Exponentially increasing competition is exposing flaws in the current system of selection for specialty training

**DOI:** 10.1016/j.fhj.2025.100487

**Published:** 2025-11-12

**Authors:** Benedict Aveyard, Stephanie Asbridge

**Affiliations:** a Clinical Education Fellow at South Warwickshire NHS Foundation Trust, Warwick, CV34 5BW, UK; b Foundation Year 2 Doctor at University Hospital Coventry and Warwickshire, Coventry, CV2 2DX, UK

**Keywords:** Selection, Specialty training, Competition ratio, Unemployment

## Abstract

Competition to get into specialty training is increasingly exponentially, exposing issues with the current selection process. Flaws include biases in portfolio scoring matrices against those at financial disadvantage, incentivising application to multiple specialties, employing solely non-clinical criteria for selection for interview, and inappropriate use of the multi-specialty recruitment assessment. Increasing competition puts greater emphasis on these components in selection, with many potential negative ramifications.

Efforts must now be made to address the flaws highlighted. We propose that this starts with incorporating clinical measures, such as multi-source feedback and clinical self-development activities, in portfolio scoring. However, an increased number of available training posts is what is ultimately needed to reduce the intense competition for jobs, take pressure off the current system, and allow its flaws to be addressed.

We are concerned that the current situation, in which competition ratios for entering specialty training are rising exponentially,^1^ is exposing flaws in the selection process. Flaws highlighted include portfolio scoring matrices that are biased against those at financial disadvantage, incentivising application to multiple specialties, use of solely non-clinical criteria for selection for interview, and inappropriate use of the multi-apecialty recruitment assessment (MSRA). We will argue that these issues are compounded by the increased competition for entering specialty training, and that this is a situation which needs urgent measures to resolve.

Portfolio requirements for getting into training include domains that are likely to incur costs to fulfil. For example, in the internal medicine training (IMT) portfolio,^2^ points are scored for having postgraduate degrees, presenting at medical meetings, publications, teaching experience, training in teaching, and quality improvement (QI) projects. Postgraduate degrees and training in teaching (such as undertaking the PGCert in medical education) come at significant cost to both time and finances. Presenting at medical meetings may incur conference attendance fees, as well as travel and accommodation costs. Evidence has suggested that a UK trainee could spend approximately £5,411 on conference fees throughout their training^3^ and this cost is only likely to increase due to inflation. The foundation programme does have a study budget that can be used for various educational activities, including conference fees, but the estimate cited takes account of this subsidisation and therefore highlights the remaining financial barrier. Such significant costs to reach benchmarks for interview ultimately give an unfair advantage to resident doctors who can afford to pay them. A doctor’s financial background should not be a factor contributing to selection: this is not fair. Medicine is already a field in which only 5% of its students are from working class backgrounds,^3^ so further selection biases at later stages of training are especially concerning and unacceptable.

Furthermore, we currently have a selection for interview process that has no weighting on an applicant’s clinical ability. While interview stages involve clinical scenarios, the current competition and lack of resource availability to interview large numbers of applicants mean that, ultimately, many are turned away from training based on solely non-clinical parameters.

During the foundation programme, trainees are allocated self-development time (SDT), which is a valuable opportunity to develop skills outside day-to-day working. An important part of becoming a well-rounded clinician is the acquisition of non-clinical skills, such as teaching, leadership, and management of services and staff. SDT can be an excellent time to develop these skills. However, we are concerned about the current imbalance in use of SDT for non-clinical learning at the expense of gaining important clinical skills and knowledge. We think that having only non-clinical selection criteria for interview will drive applicants to spend a disproportionate amount of their SDT, and their own time, working towards non-clinical learning only. For example, the IMT applicant scoring matrix^2^ comprises solely non-clinical parameters as listed earlier, with no measure of clinical acumen. This risks disincentivising trainees from seeking important clinical learning opportunities during SDT. For instance, there are skills expected of registrar-level trainees that are not performed frequently, such as pleural aspiration (for non-respiratory physicians). This skill is sometimes required out of hours in an emergency, commonly expected to be performed by non-respiratory specialists.^4^ A nationwide survey among intensive care trainees suggests variable but overall low confidence currently in performing pleural procedures.^4^ If more SDT was utilised to practise low-volume but important clinical skills such as this, we may have a more skilled, safer, and ultimately more well-rounded cohort of doctors. Redressing the balance of criteria in selection for interview to include both clinical and non-clinical parameters is needed in order to encourage and incentivise proactive clinical learning in this way.

If selection for interviews had at least one component that focused on the clinical practice of medicine, this would help restore balance. With limited resources available for selection, and the subjective nature of assessing applicants’ clinical acumen, this is a difficult task. Clearly, there are questions regarding how clinical criteria for selection would be validated and utilised, especially given such large number of applicants. Resolving these issues would therefore require consultation with various stakeholders, and we alone do not propose to have all the solutions.

Awarding points in selection for interview for courses that develop clinical skills, such as point-of-care ultrasound courses, could be a way to objectively measure and incentive engagement with clinical learning alongside non-clinical projects. An important caveat to rewarding attendance at these courses would be the need for equitable funding in order to avoid disadvantaging applicants with less financial means. However, it is important to remember that existing criteria, such as postgraduate qualifications and publications, harbour similar unresolved issues such as insufficient funding and accessibility, as discussed previously.

Another clinical criterion could be based on multi-source feedback (MSF), with candidates given numerical scores in various domains. For example, these could include clinical knowledge, clinical skills and communication, as well as some professional criteria such as time management and organisation. We recognise that using MSF incurs problems with validation, which may be difficult to resolve fully. However, evidence shows that appropriate validation is also an active issue for current non-clinical criteria for IMT interview selection.^5^

There is currently a rapid rise in scientific fraud, with some journals accepting papers for significant fees, with minimal quality assurance, or even guaranteed publication.^5^ It is common for medical residents in China to be involved in paper fraud, which has even been described as a possible ‘new norm’ among trainees globally.^5^ Given that having a first-author publication yields the most points out of all criteria for IMT selection for interview,^2^ and is therefore intensely incentivised, it may not be surprising if this finding was replicated across UK resident doctors. Equally, such fraud is not something that assessors may be able to police effectively, especially given that only a proportion applications require evidencing as part of a randomised audit. Clearly, candidates who commit scientific fraud also commit a serious breach of probity, but on paper may present with strong applications. As well as potentially selecting for dishonesty among trainees, scientific fraud has wider implications for patient safety, for instance in risking advising inappropriate practice. Training doctors with skills in academia is to be encouraged and celebrated, and selection criteria should reflect this. However, incentivising publications so intensely ultimately risks pushing trainees with limited interest in clinical academia towards unscrupulous methods in order to make successful applications.

A further worry for doctors at present is the risk of unemployment. There are concerns that approximately 20,000 doctors could miss out on specialty training posts this year.^1^ Anecdotally, many of our resident doctor colleagues are very anxious about whether they will have a job next year. This anxiety is reflected in a publication from the Royal College of Physicians highlighting the risks of unemployment post-foundation training, and the distress that this can cause.^6^ Evidence suggests that economic stress such as this is associated not only with mental health decline and burnout, but also with impaired clinical decision making, thus risking patient safety.^3^ To mitigate these risks, each applicant is applying to an increasing number of specialties each year^7^ – a trend which we have seen reflected among our colleagues – including specialties that they would not particularly want to practise, out of fear of unemployment. This creates a risk that resident doctors will accept training posts in specialties in which they are not interested or perhaps not well-skilled, purely to secure a job. Downstream, this could result in senior doctors who are not enthusiastic about, satisfied or fulfilled by their specialties, and therefore at higher risk of burnout or of leaving medicine.^8^

Clearly, there is need for more training posts to mitigate fears of unemployment. Given the rise in applications from international medical graduates (IMGs) since the Resident Labour Market Test was removed on 20 January 2021,^9^ the government is considering prioritising UK graduates for jobs within the UK.^1^ This would bring the policy in line with most other countries.^9^ Such a measure would certainly help reduce competition, but is not without controversy and complex legal challenges, and does not address the fundamental shortage of training posts available. There is already a deficit in the number of consultant physicians, and yet demand for senior clinicians is only likely to increase given the increasingly ageing and multi-morbid UK population.^6^ If we were to prioritise UK graduates, this could discourage IMGs from working within the NHS, and at a time when we are already short of doctors, are we really in a position to be turning them away? We need to train more consultants, requiring additional training posts to reduce bottlenecks at this career stage. An alternative way to reduce competition ratios without selecting doctors based on their country of training would be to mandate experience working within the NHS prior to starting a training programme. This would ensure that those in training were familiar with how the NHS works, and therefore more effective clinicians. However, this could risk IMGs being forced into locally employed posts without significant career progression. This is a very complex issue, with many legal and ethical ramifications. Ultimately, the most important solution for issues with both intense competition and shortage of senior doctors is to increase number of training positions.

Many training pathways, for example core surgical training (CST), anaesthetics and acute care common stem (ACCS), use an exam called the multi-specialty recruitment assessment (MSRA) to select for interview. Psychiatry and general practice (GP) training use the MSRA alone to decide on applicant recruitment. The MSRA is a computer-based examination aimed at assessing an applicant’s clinical knowledge via multiple choice questions, as well as a situational judgement test. While this is a validated tool as part of the selection process for GP, psychiatry and radiology, this is in the context of a whole assessment process rather than as a standalone tool.^10^ Further, the MSRA lacks validity as an assessment tool for other specialties.^10^ For example, the MSRA exam was designed to select for GPs and therefore spans the range of medicine. How much benefit will future anaesthetists derive from revising emergency contraception as part of MSRA preparation? How appropriate is it for their performance in this exam to determine their selection for interview? Given concerns regarding the use of the MSRA as such a significant part of selection across a range of specialties, we propose that this examination is only used where its use has been validated as a selection method.

To summarise, the rising competition for training posts is exposing and potentiating the flaws in the current methods of selection. Portfolio scoring metrics show bias against those at financial disadvantage. Intense competition drives and incentivises resident doctors to apply for more specialties than those in which they are genuinely interested. There is a concerning lack of clinical aptitude criteria for selection for interview, potentially biasing recruitment against skilled clinicians in favour of those with more audits under their belts. Further, intensely incentivising publications may even be contributing to scientific fraud, with potential effects on patient safety. Evidence shows that the MSRA is currently being used outside its scope of validity and may therefore not be selecting the most appropriate candidates for certain specialties. Significant changes to the application and selection system are clearly required, for instance increasing the number of training posts available and necessitating some experience of working in the NHS in order to apply for specialty training. A crucial first step is addressing the biased and overwhelmingly non-clinical portfolio scoring, which is one of the first barriers to many strong applicants entering training. Incorporating clinical measures would shift the weighting to also consider clinical ability and drive further learning in this domain.

## Funding

This research did not receive any specific grant from funding agencies in the public, commercial or not-for-profit sectors.

## CRediT authorship contribution statement

**Benedict Aveyard:** Writing – review & editing, Writing – original draft, Conceptualization. **Stephanie Asbridge:** Writing – review & editing.

## Declaration of competing interest

The authors declare that they have no known competing financial interests or personal relationships that could have appeared to influence the work reported in this paper.
